# Sock and Environmental Swabs as an Efficient, Non-Invasive Tool to Assess the *Salmonella* Status of Sow Farms

**DOI:** 10.3390/ani13061031

**Published:** 2023-03-11

**Authors:** Kathrin Lillie-Jaschniski, Christoph Wähner, Miriam Viehmann, Silke Hauf, Christina Gale, Judith Rohde, Isabel Hennig-Pauka

**Affiliations:** 1 CEVA Tiergesundheit GmbH, 40472 Düsseldorf, Germany; 2 Tierarztpraxis Cappel, Hallerstr. 142, Öhringen-Außenstelle, 74613 Schrozberg, Germany; 3 Tiergesundheitsdienst Bayern e.V., Senator-Gerauer-Str. 23, 85586 Poing, Germany; 4 CEVA Animal Health Ltd., Amersham HP7 9FB, UK; 5 Institute for Microbiology, University of Veterinary Medicine, Hannover, Foundation, Bischofsholer Damm 15, 30173 Hannover, Germany; 6 Field Station for Epidemiology, University of Veterinary Medicine, Hannover, Foundation, Buescheler Straße 9, 49456 Bakum, Germany

**Keywords:** *Salmonella*, sock samples, environmental swabs, diagnostics, boot swabs

## Abstract

**Simple Summary:**

*Salmonella* is an important pathogen in both livestock and humans, therefore much research has been conducted aiming to improve control methods and consequently reduce prevalence. *Salmonella* monitoring systems have been implemented in swine slaughter plants across Germany to test meat juices or blood of finisher animals being processed. The farms are placed into categories according to the level of *Salmonella* positivity, which then dictate actions required to be taken by the farmer as well as any price penalties applied to the meat sold. Since the implementation of this monitoring system, the distribution of farms across categories has not drastically altered, indicating that the national *Salmonella* situation is not necessarily improving. More action seems to be required at the breeding and multiplying levels of production rather than simply monitoring the finisher farms. Improving the diagnostic methods at these early stages of production in sows and nursery pigs, and consequently the control of *Salmonella*, would aid improvement of the *Salmonella* situation across the whole pig stock across Germany, including finishing farms. Sock and swab sampling has been demonstrated as a simple, cheap and effective sampling method for other enteric pathogens and therefore may be a useful tool for the diagnosis of *Salmonella* on swine farms. Availability of an easily accessible diagnostic method for *Salmonella* may increase investigations into the pathogen on sow farms and consequently improve the control methods implemented and reduce *Salmonella* positivity at slaughter.

**Abstract:**

Salmonellosis is the second most reported gastrointestinal infection in humans after campylobacteriosis and a common cause of foodborne outbreaks in the European Union (EU). In addition to consumption of contaminated animal-based foods, such as poultry, beef and eggs, pork is an important source of human salmonellosis outbreaks; therefore, *Salmonella* (*S*.) control should start in the early stages of pig production. To be able to implement effective control measures to reduce the risk of pigs being infected by *Salmonella*, it is important to identify the serovars circulating on farm within the different stages of production, including as early as sow and piglet breeding. The aim of the present study was to assess the *Salmonella* status of sow farms either producing their own finishers or delivering piglets to fattening farms with a known high serological prevalence identified within the QS *Salmonella* monitoring system. Overall, 97 (92.4%) of 105 investigated piglet-producing farms across Germany tested positive in at least one sample. *Salmonella* was detected in 38.2% of the sock and 27.1% of the environmental swab samples. *S*. *Typhimurium* was the most frequent serovar. In conclusion, sock and environmental swab samples are well suited for non-invasive *Salmonella* detection in different production units in farrowing farms. To establish a holistic *Salmonella* control program, all age classes of pig production should be sampled to enable intervention and implementation of countermeasures at an early stage if necessary.

## 1. Introduction

*Salmonella* (*S*.) is a Gram-negative, facultative anaerobic rod-shaped bacterium, which can cause diarrhea and septicemia [1]. More than 2500 serovars [2] with different virulence levels and host adaptations exist [3]. According to the EFSA European Union One Health 2020 Zoonoses Report published in 2021, *Salmonella* remains one of the most frequently reported causative agents of foodborne outbreaks [4]. The most common sources of salmonellosis outbreaks are eggs, egg products and pig meat, with the most commonly involved serovar being *S.* Enteritidis, followed by *S. Typhimurium* and its monophasic variants [5]. Due to the leading role of pigs as a source of *Salmonella* introduction into the food chain, different control programs for *Salmonella* in pigs have been implemented in Europe [6]. A huge amount of research has been conducted worldwide, focusing on the epidemiology and control of *Salmonella* in pigs [7,8,9,10,11]. The EFSA Annual Report 2008 highlighted the impact of the *Salmonella* status of breeding herds, demonstrating that countries with a low *Salmonella* prevalence in breeding herds tended to have relatively lower prevalence in slaughter pigs compared to countries with high breeding herd prevalence [12]. *Salmonella* positivity in fatteners was also impacted by the prevalence of *Salmonella* in the breeding herd [12,13], emphasizing the importance of the control of breeding herds supplying fatteners.

*Salmonella* diagnosis is often very challenging due to shedding pattern variation between different serovars and differences in the infectious dose received by the animal [14,15], as well as the intermittent shedding of carriers [16]. In Germany, a non-governmental serological *Salmonella* monitoring system in pigs intended for human consumption was introduced by the German Quality Assurance System (QS) (https://www.q-s.de/en/, accessed on 3 December 2022) in 2002 [17]. The *Salmonella* status of a fattening farm is determined quarterly by testing either meat juice/blood samples taken at the slaughterhouse or blood samples taken from fatteners up to 14 days before slaughter [18]. Every year, 60 samples per farm (15 per quarter) are examined with one of three validated *Salmonella* ELISA test kits, using a standardized cut-off at OD 40% [17]. Based on the antibody determination of these samples, the farms are divided into three categories based on the risk of *Salmonella* introduction into the slaughterhouse: category one (low risk) 0–20% positive samples; category two (medium risk) 20–40%; and category three (high risk) more than 40% positive samples. Farms in category three must identify sources of *Salmonella* entry and initiate control measures in consultation with their farm veterinarian. Since the introduction of *Salmonella* monitoring, a decrease in category-three pig farms has been observed from 5.8% in 2005 to 1.6% in 2020 [19].

Various studies have shown that direct contact with infected animals and the introduction of already infected piglets into nurseries [20,21] and fattening units [8,11,13,21,22] present a greater risk of infection than contaminated feed [23] or the environment. Therefore, control methods should aim to reduce the presence of *Salmonella* in all early stages of production (gilts, sows, piglets) to help to decrease the risk of infection in fatteners and consequently the risk of introduction of the pathogen into the food chain [13,24,25]. Often, animals on *Salmonella*-infected farms do not show clinical signs, and therefore it is important to use a sensitive sampling method to identify infected breeding herds and prevent purchases of asymptomatic carriers [22,26,27]. Additionally, the sampling method must be practically and economically feasible to ensure it can be used on a large scale. Although serology on sows could be valuable to identify the *Salmonella* prevalence within a sow herd [28,29], blood samples do not give information about the serovars involved or the spread of the pathogen on the farm, in addition to having possible welfare implications. For direct detection of *Salmonella* on-farm, bacteriological examination or direct PCR methods using fecal samples and different types of environmental samples have been described, including surface wipes and sock swabs [9,10,11,22,30]. However, large numbers of fecal samples are required to detect low shedding rates within groups of animals [8,11,22,29].

Sock and environmental swab samples have already been described as a reliable diagnostic method in nursery pigs, guiding clinical decisions for treatment and prevention [31], and have been proven to be a suitable method for monitoring farms and detecting *Salmonella* in poultry [32,33,34]. Furthermore, in a study including 101 fattening and 41 farrow-to-finish farms in the north-west of Germany, environmental sampling was proven to provide an adequate basis for *Salmonella* control in farms with a high seroprevalence. Significantly more areas tested positive compared to farms with a low seroprevalence [30].

In contrast to individual fecal samples, the sock and swab method does not give information about the *Salmonella* prevalence within a group of animals. However, using one swab and one sock within a compartment means that only a small number of samples are required per farm. Low shedding rates of sows, shown to often be below 10%, would require a high number of individual fecal samples. To detect one shedding sow in a 1000-sow herd with 10% *Salmonella* prevalence with a 95% confidence level, 28 samples would be necessary [35,36]. In a longitudinal follow-up trial in a *Salmonella* control program in the UK, 60 individual samples in each epidemiological group (dry sows, lactating sows, weaners, etc.) were required each visit to address a 5% prevalence presuming 100% sensitivity [24]. Using pooled fecal samples lowers the number of samples needed [37,38]. In another longitudinal approach, sock samples led to a higher sensitivity than pooled fecal samples [9]. Environmental swabs were found to be an ideal method for identifying residual *Salmonella* in areas which are usually not accessible by animals within the compartments, such as, the undersides of troughs and window sills covered with dust [30].

The aim of the present study was to assess the *Salmonella* status of sow farms either producing their own finishers or delivering piglets to fattening farms. All downstream fattening units had a known high serological prevalence identified within the QS *Salmonella* monitoring system. The upstream sow herds were selected for sampling depending on the serological status of the associated fattening herd. As only some federal states in Germany are offering facultative *Salmonella* monitoring of sow farms, the sow farms in this study were sampled for *Salmonella* for the first time. Previous studies have shown the viability and sensitivity of sock and environmental swab samples for individual farms with different serological statuses [9,11,30,39,40]. However, this large-scale study aimed to determine whether sock samples from pens and environmental swab samples from reachable surfaces are suitable to identify areas contaminated with *Salmonella* in sow farms and the associated nursery, which are linked to fattening farms with a high serological *Salmonella* prevalence. Additionally, it was assessed whether a minimum of 16 samples per farm is a suitable number of samples to detect *Salmonella* on-farm. As in a recent publication, it was concluded, that sock sampling alone would be sufficient to determine the status of a herd [9], within this trial, we investigated, if both types of samples are needed to give a good overview of the farm. Additionally, it was aimed to identify the serovars present on the farms to be able to implement appropriate control measures.

## 2. Materials and Methods

### 2.1. Study Farms

A total of 105 farms across Germany, including two farrowing farms, 71 farrow–nursery farms and 32 farrow-to-finish farms with sow numbers ranging from 50 to 10,000 were sampled within this study. The evaluation period was from January 2015 to July 2021. All sow farms selected for the study were linked to fattening units with a known high *Salmonella* (*S*.) seroprevalence.

### 2.2. Sampling

The number of sock and environmental swab samples taken on each farm depended on the size of the farm and the production cycle. To cover all production sites, one sock sample and one environmental sample each were taken in two farrowing rooms (around farrowing and shortly before weaning), in the insemination room, in gestation rooms (pens with newly grouped sows), the gilt quarantine room and in three rooms in the nursery (beginning, middle and end of nursery), resulting in a minimum of 16 samples taken per farm. In farms containing more than one compartment filled with the respective age group/stage of production, all rooms belonging to these ages were sampled. Empty, cleaned and disinfected compartments already prepared for the arrival of new pigs were sampled if accessible during the farm visits. This was performed in farms using a routine protocol for cleaning and disinfection, including mechanical removal of dirt before soaking with water and cleaning with a high-pressure washer followed by drying and disinfection. To increase the probability of finding pigs shedding *Salmonella*, groups were sampled preferentially after a stressful period, e.g., after they had been moved from the mating pen to a holding pen.

**Figure 1:**
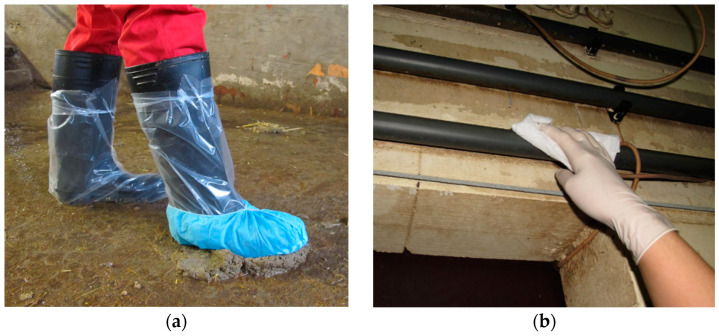
(**a**) Sock sampling; (**b**) Environmental swabs.

All materials used for the sampling were dry, clean and individually packed by Sterimatic, Stroud, UK. The sample kits contained one pair of non-sterile gloves (lightly powdered latex examination gloves, Covetrus Germany), a dry synthetic dust cloth (Swiffer^®^, Procter & Gamble Service GmbH, Crailsheim, Germany), one pair of plastic overshoes (PE boot cover transparent, WIROS Wilfried Rosbach GmbH, Willich, Germany) one sock (“Hygostar” polypropylene nonwoven overshoes, Franz Mensch GmbH, Buchlohe, Germany) and two plastic bags. Sampling was carried out by one of four trained and experienced people. To prevent cross contamination, a new pair of gloves was used for each sample collection. After sampling, the socks and swabs were placed into individual plastic bags, which were firmly closed and individually marked. When collecting the sock sample, each boot was first covered with the plastic overshoe before entering the pen. The sock was placed over one plastic shoe to collect fecal material from the floor of each pen within the sampled compartment (Figure 1a). All pens within a sampled compartment were sampled in a standardized way. As much fecal material was stepped in as possible within each pen. It was walked along the walls, into the corners, around the water supply and the trough. While sampling the pens, it was important not to contact the walkways to ensure the sample only reflected the shedding of the animals within the pens. In the cleaned and disinfected compartments, the focus was on stepping into obvious dirty spots or, if present, into dead flies and rodent feces. One environmental swab was used per room to sample sedimented dust on water pipes and feeding lines (a minimum of 3 m at different locations) (Figure 1b), as well as walls above the belly button height, surfaces of air inlets, ventilation systems and window sills.

All samples were shipped without extra cooling by an overnight courier to the laboratory.

### 2.3. Bacteriology

Socks and environmental swabs were examined bacteriologically at the Institute for Microbiology at the University of Veterinary Medicine in Hanover (Stiftung Tierärztliche Hochschule Hannover, Institut für Mikrobiologie, Bischofsholer Damm 15, Gebäude 126, 30173 Hannover, Germany). All samples were enriched non-selectively in 225 mL of buffered peptone water to yield a tenfold dilution (BPW; Oxoid, Thermo Fisher, Wesel, Germany) and incubated for 18 h at 37 °C. From this non-selective pre-enrichment, 1 mL was transferred to 8 mL of tetrathionate brilliant green bile broth (TBG; VWR, Darmstadt, Germany) and 0.1 mL was transferred to 10 mL of Rappaport-Vassiliadis Soy Broth (RVS; Oxoid, Germany). Both tetrathionate brilliant green bile broth and Rappaport-Vassiliadis Soy Broth acted as selective liquid media. All samples were incubated for 24 h at 42 °C and then streaked on Oxoid Brilliance Salmonella Agar. The plates were inspected for growth of typical purple colonies after 24 h at 37 °C. One typical colony per sample was subcultured on non-selective sheep blood agar (Oxoid, Germany). Colonies were then submitted to an in-house PCR that identified *S*. *Typhimurium* (STM) (including the monophasic variant of STM (mSTM)), *S*. Enteritidis (SE) and *S.* Dublin. Other serovars produce a *S. enterica* ssp. *enterica*-specific and/or a genus-specific signal in this multiplex PCR [41]. Further characterization via slide agglutination for a selection of relevant O- and H-specific antigens (Sifin Antisera, Berlin, Germany) was completed if isolates were only identified as *S*. *enterica* ssp. *enterica* and/or *S.* genus by PCR. These antisera included anti-O4, anti-O5, anti-O9, anti-group C, anti-group E; anti-Hi, anti-H2, anti-Hf, anti-Hg, anti-Hm, anti-Hp, anti-Hq, anti-Hs and anti-Ht). A farm was classified as *Salmonella*-positive if at least one sample tested positive. For farm analysis, the samples were grouped according to their positivity for STM, *Salmonella* Enteritidis (SE), *S.* Derby (SD) or for other serovars. The full identification process was performed only for STM, SE and SD, whereas the other isolates were only identified within their serogroup.

### 2.4. Statistical Analysis

The data were tested pairwise between the groups with a two-sided Wilcoxon–Mann–Whitney U-Test, alpha = 0.05. Tests were performed per region (East/West Germany) and per farm size (1–299 sows, 300–999 sows and more than 1000 sows) and additionally considering the difference in positivity within each area of the farm (farrowing, nursery, other areas). For the binominal distribution of samples within each area, the confidence interval (CI) was set at 95%. To evaluate a possible difference in positivity of sock and environmental swabs, a McNemar test was used, with *p* < 0.05 showing a statistical difference between frequencies of positive samples between the two types of samples. Additionally, the Kappa Index was calculated to obtain a measure of the agreement of these two methods (<0.01: no agreement; values between 0.1 and 0.4: weak agreement; values between 0.41 and 0.60: clear agreement, values between 0.61 and 0.80: strong agreement) [42]. The analysis was performed with the validated program Testimate Version 6.5 from IDV Datenanalyse und Versuchsplanung, Gauting, Germany.

## 3. Results

In total, 105 farms were included in the study. At farm level, 97/105 farms (92.4%) from all over Germany tested positive for *Salmonella* in at least one sample. Evaluating samples geographically showed that 27/30 (90%) of the farms in eastern Germany tested positive and 70/75 (93.3%) of the farms in western Germany tested positive.

**Figure 2:**
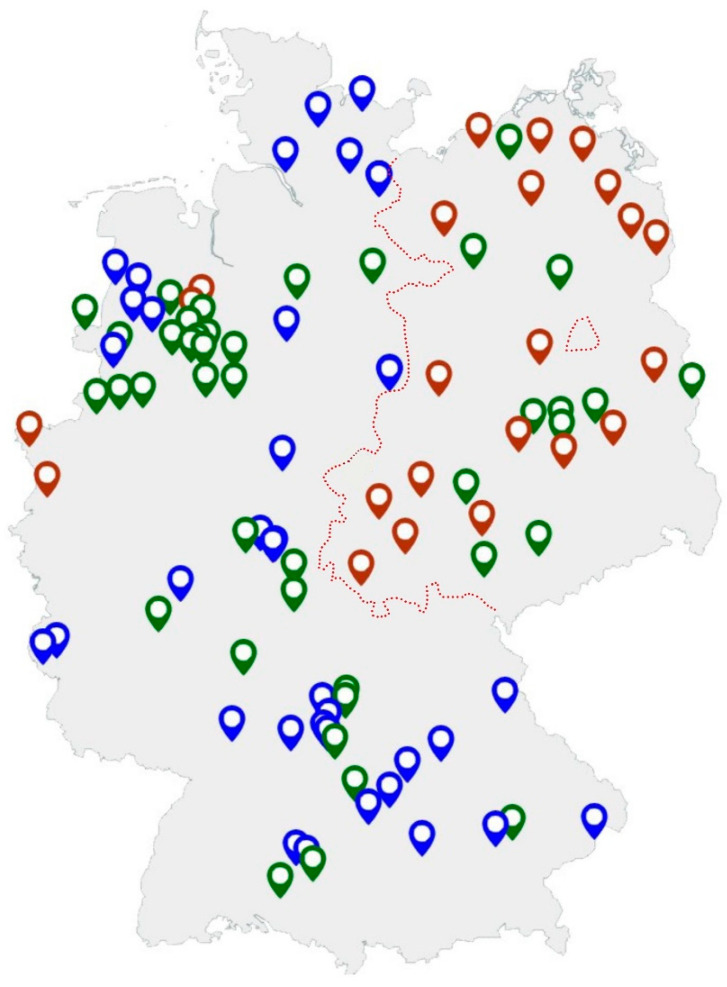
Location of tested sow farms (blue: farms with up to 299 sows, green: farms with 300–999 sows, red: farms with >1000 sows). The border of former East and West Germany until 1989 is indicated by the red line.

In summary, 36/37 (97.3%) farms keeping one to 299 sows and 36/42 (85.7%) farms with 300–999 sows tested positive. Nearly all farms (22/23) with over 1000 sows were found to be positive (95.5%). For three farms, all of which were positive for *Salmonella*, no information about the farm size was available. All farms with <300 sows were located in western areas of Germany (*n* = 37), while no farms of this size were present in eastern areas of Germany. There were no statistical differences between farm size, region and positivity for *Salmonella*. Figure 2 gives an overview of the location of the tested farms.

The number of samples taken per production area is depicted in Table 1. In total, 2355 environmental and sock swabs with an average of 22 samples/farm were analyzed (detailed information about the number of samples taken per farm is shown in the Supplementary Material Table S1). In total, 779 samples were identified as positive (33.1%). Most samples (*n* = 970) were taken from the nursery, followed by 662 samples from the farrowing area. A further 452 samples were taken from other areas, including gestation, gilt quarantine and insemination areas. Cleaned and disinfected compartments and boot washers were available on 23 and 15 farms, respectively.

**Figure 3:**
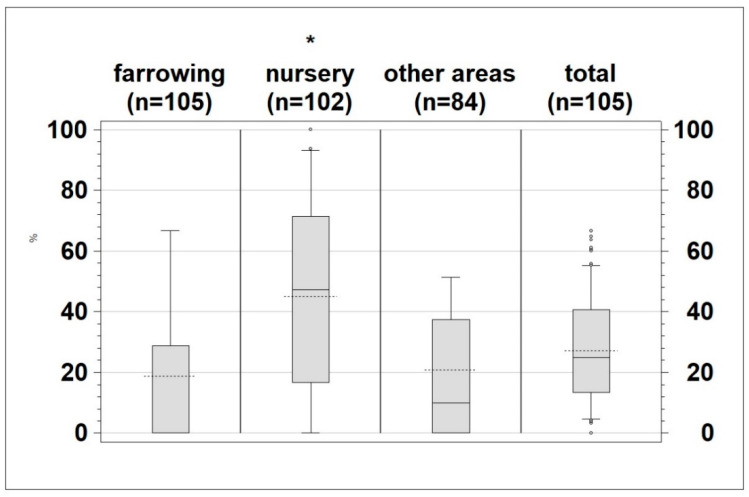
Percentage of positive samples per sampled area (farrowing, nursery, other areas and total) * *p* < 0.0001, dotted line: mean; whiskers: 10th and 90th percentile.

The proportion of positive samples (mean 27.2%) at herd level is shown in Figure 3. The number of samples that tested positive within the different production areas differed for the individual farms. Nevertheless, a significantly higher (*p* < 0.0001) proportion of samples from the nursery were positive for *Salmonella* (mean 45%) compared to those taken from farrowing (mean 18.7%) and other areas (mean 20.86%) (Figure 3).

**Figure 4:**
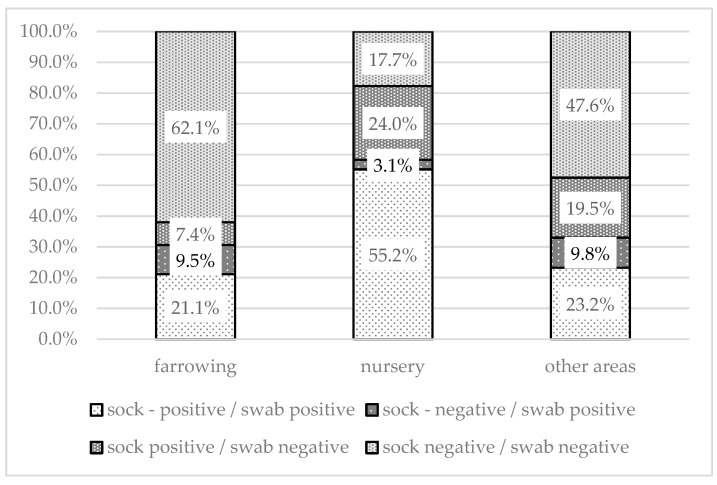
Comparison of positivity of sample by method (sock swab/environmental swab) within each sampled area.

The proportion of different combinations of positive or negative swabs and environmental samples varied for different sampling areas (Figure 4). Both positive sock and swab samples were found most often in the nursery (55.2%). A combination of positive swabs and negative socks was the least commonly recorded. Negative results for both sock and swab samples were highest in farrowing areas (62.1%). In 24% of the nurseries, only the socks were positive; this was significantly higher (*p* < 0.001) than the proportion of nurseries in which only swabs were positive (3.1%). The results of the statistical analysis are shown in Table 2.

**Figure 5:**
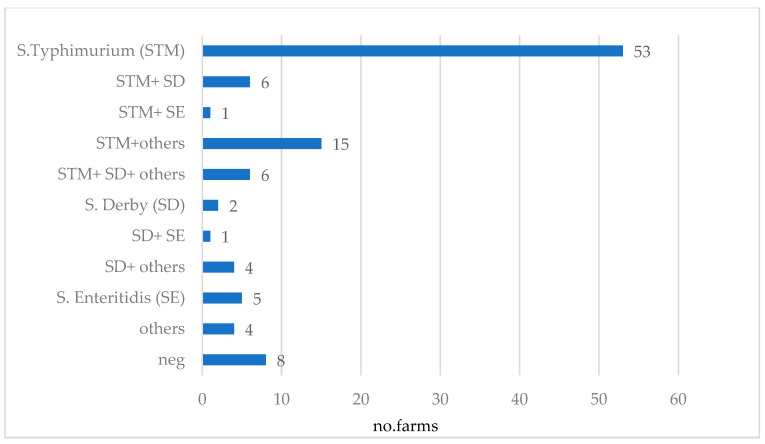
Combinations of *Salmonella* serovars detected in 105 German sow farms via bacteriological examination of sock and environmental swab samples (others: *S.* group B (other than STM and SD), *S.* group C and *S*. group E and non-typable isolates given the limited set of sera implemented in this study).

The distribution of detected serovars across the 105 farms is presented in Figure 5. *S*TM was found on 77.1% (*n* = 81) of the farms and in 50% (*n* = 53), it was the only serovar detected. Detection of STM together with SD occurred in 11.4% (*n* = 12) of farms, while the detection rate of STM combined with other serovars was 15.2% (*n* = 16). SD could be detected on 18.1% (*n* = 19) of the farms, and in 1.9% (*n* = 2) of cases, it was the only serovar detected. *Salmonella* Enteritidis (SE) alone occurred on 4.8% (*n* = 5) of farms, and in combination with other serovars in 1.9% (*n* = 2) of farms. Positivity for other serovars alone was reported for 3.8% (*n* = 4) of farms, and on 7.6% (*n* = 8) of farms, no *Salmonella* could be detected. A complete overview of the serovars and serogroups detected on each farm is provided in the Supplementary Material Table S1.

Evaluation of individual farms and their different production areas shows that 97/105 (92.3%) were positive for at least one serovar. While in 9 farms (8.6%) only one serovar was found in other areas, and nursery and farrowing were negative for *Salmonella*, in 19 farms (18.1%), only the nursery was positive and one serovar was identified. On 31 (29.5%) farms, different serovars were detected in different positive areas of the farm. (Supplementary Material Table S1).

## 4. Discussion

This study aimed to identify *Salmonella* (*S.)* on 105 piglet-producing farms in Germany, which may be the source of *Salmonella* introduction into the linked fattening farms. The farms involved were all delivering pigs to fattening farms with high serological prevalence, as identified via the obligatory German serological monitoring system [17], or were experiencing clinical issues due to *Salmonella.* These included severe, hard-to-treat clinical signs, reduced daily weight gain or an unspecific increased number of losses and runts [1]. Before sampling, farm *Salmonella* status was unknown. This initial sampling was carried out to gain insights into the groups of animals shedding *Salmonella* and the environmental load. Identification of serovars present on the farms was also an aim of the study. Sock and environmental swab samples were used to identify *Salmonella* serovars present on farm, with sock sampling being reflective of bacteria shed by the animals [9] and environmental swabs giving an overview of the *Salmonella* contamination in the environment [30]. We also aimed to identify the areas of production with the highest shedding and environmental pressure, as shown by the number of positive samples, and determine if both sample materials are really needed to identify the production areas at risk within a farm.

*Salmonella* was detected in 92.4% (*n* = 97) of the studied farms, with a farm being classified as positive when just one sample was positive for *Salmonella*. In our study, the chance of identifying *Salmonella* was independent of the farm size, which is contrary to other publications where a relationship between the *Salmonella* positivity and the size of the farm or group size was reported [43]. Previously, STM shedding of breeding pigs was found to be associated with herd size and the number of pigs in the pens [25]. In contrast, we found no significant difference regarding the positivity within each production area within the farms when comparing farm size and region. The high positivity rate and the lack of variation in farm size and region within this study could be due to herd selection being based on linkage to fattening farms with a known high serological prevalence or the presence of clinical signs with suspicion of *Salmonella* involvement. This is supported by previous authors demonstrating the direct link between serologically positive fatteners and the *Salmonella* prevalence of breeding herds [12,13].

The eight farms that had negative results might have truly been negative; however, a false negative result may occur due to either a low sample size that was not sufficient to detect a very low prevalence or the introduction of *Salmonella* into the fattening farms via other routes, such as feed, rodents or other piglet origin [23,25,44]. Another possible explanation could be infection of animals with a host-adapted *Salmonella*, mainly *S*. Choleraesuis (SCS), which may have led to a strong immune response and therefore a high serological prevalence in fatteners. Unlike most of the other serovars, SCS is seldom detected in the environment [1]; however, the pathogen can be found during necropsy using suitable tissue for isolation, including that from the lungs, especially the cranioventral portion of the caudal lobe, mesenteric lymph nodes, spleen, liver, and kidneys [45]. As a next step, farms testing negative should be sampled a second time to validate the result. If this result is confirmed, in these cases the risk of infected piglets being introduced into fattening farms is very low. Thus, the hygienic measures in these fattening farms seem to have most impact on the *Salmonella* status of their fattening pigs before slaughter.

The highest number of positive samples (*p* < 0.0001) was found in the nurseries. This supports previous findings [8,11,29] reporting the highest detection rates in the nursery. Especially important are the findings of Bernad-Roche et al. in 2021 [21], where they demonstrated that the serovars detected in weaners and within a given nursery remained the same over a long period of time (>150 days), and were found even later in gilt rearing units. The high number of positive samples in the nursery in our study supports the hypothesis that these piglets are a source of *Salmonella* for the linked fattening farms, particularly as they can become subclinically infected and act as active carriers of *Salmonella* [21].

Our study underlines the risk of suckling piglets becoming infected in the farrowing units. The mean of samples in the farrowing areas that were positive for *Salmonella* was 18.9%, with a maximum of 70% of samples positive in some farms, which is much higher than reported by Hollmann et al. 2022, who in the peripartal period only found a few boot swabs positive for *Salmonella* [11]. Other authors also reported prevalence below 10% in three Belgian sow farms using individual fecal samples [22], similar to another study where just a low amount of environmental samples taken in 12 *Salmonella*-suspected identified farms were positive in culture [46]. Our data underline, that environmental sock and swab samples taken in the farrowing area can indicate shedding of sows, shedding of previously infected suckling piglets or a contaminated environment. It has been shown that weaning piglets can be infected and positive in the mesenterial lymph nodes [20,21]. Therefore, the farrowing area should be sampled in a thorough manner, especially the environment, to assess the risk for the first infection of piglets as early as the suckling period. Even if vaccination is successful at reducing shedding of the sows [24,47], farrowing houses are difficult to clean and disinfect [48] and therefore can be a source of constant reintroduction of the pathogen into the nurseries.

In 23 farms it was possible to take samples in cleaned and disinfected compartments. In 14 (60.9%) of these farms, allegedly cleaned and disinfected areas were positive for *Salmonella*, leading to 35.9% (*n* = 23) positive samples. Sampling of some of these compartments led to detection of visible contamination (old feed in feeders, droplets of feces in the corners or dust in the water/feed pipes), which indicates the need for improvement of hygiene measures. Residual dirt on drinkers and feeders in cleaned and disinfected compartments have been shown to be a source of reinfection of pigs [49]. In poultry, there is evidence for contaminated feeders and drinkers being reservoirs for *Salmonella* in production systems [50,51]. Thorough cleaning prior to disinfection to remove any organic matter is vital in ensuring the effectiveness of the disinfectant is not reduced [52]. Non-adequate room temperature, especially one that is too cold, and the pH of the solution prepared for disinfection can affect the potency of a disinfectant [53]. It is important to (i) ensure proper drying out of a room before disinfection, (ii) ensure compatibility of used detergents and disinfectant and (iii) ensure the correct final concentration of the disinfectant [53].

In pig production, and mainly in nursery and fattening, similar to poultry production, “all-in/all-out” management is commonly used to break infection chains and minimize the risk of young animals being infected by older animals [54,55]. Walkways and any crossing of paths on walkways can also have an important role in transmission of *Salmonella* between different age groups and production areas. In this study, the walkways in 38 out of 74 farms, and the boot washers in 10 of 15 farms tested positive. As *Salmonella* can be sustained for up to 50 months in the environment, e.g., in dust [56], and up to 24 weeks in manure [57], reinfection by contact with contaminated walkways is a high risk which must be addressed in *Salmonella* control programs [9].

The findings of this study can support decisions for the best sampling strategy in a sow farm. Whereas other authors concluded that sock samples would be a good sampling strategy as a standalone method [9,11], the results of our study indicate that, besides the nursery, where sock samples were positive significantly more often than environmental swabs, both samples should be taken at each sampling point. Although the statistical analysis for the farrowing area showed that there was strong agreement between the two methods, indicating one of the methods alone would be enough, in almost 10% of cases (9.5%), a positive result would have been missed had no swab been taken. Therefore, we conclude that in farrowing, the use of both methods is advisable. The same can be recommended for the other areas. This clearly demonstrates the importance of assessing environmental contamination in these areas with a swab, as well as the impact of shedding animals, by using a sock sample [27]. Acids in the feed and specific feeding strategies, including an increase in particle sizes, as well as vaccination against *Salmonella*, can reduce *Salmonella* shedding [58,59] and might consequently decrease the detection rate of *Salmonella* in feces and thus in sock samples. Especially in these cases, environmental swabs are extremely important for detecting environmental contamination (*Salmonella* reservoirs) and therefore potential risk of reinfection.

To implement sustainable *Salmonella* programs within a production chain, it is important to assess the serovars involved. In this study, 77.1% of the farms were positive for STM followed by 18.1% for *S.* Derby, either alone or in combination with other serovars. These findings reflect the situation found in European pig production over the last few years [60], considering STM is currently the most frequently reported serovar isolated from pigs [26,27,61] and also the most relevant *Salmonella* reported in human cases related to pork or pork meat products [5]. In 35% of the farms (*n* = 37), more than one serovar could be detected. Within the serogroups C, D and E, no further analysis on the individual serotype was performed. These serogroups include serotypes such as *S.* Infantis, *S*. Rissen, *S.* Ohio and *S.* London, which are also regularly detected either on-farm [21,22,62] or in lymph nodes at slaughter [5,20,21]. Therefore, a conclusion on the exact number of different serovars present on each farm and within each production area cannot be given. In recent studies, several different serotypes within a serogroup were detected on one farm [20,21,24]. SCS, also belonging to serogroup C, is mainly found in the organs of acutely diseased pigs, and is not commonly found in the environment [1,45]. Therefore, no further analysis to identify SCS was performed within this study. On one of the farrow-to-finish farms that tested negative in socks and environmental swabs, further examinations of acutely diseased pigs showed that SCS could be found from *Salmonella* enrichment of organs (spleen, kidneys, lung and liver). The farm did not observe salmonellosis outbreaks but remained in category three within the QS *Salmonella* monitoring system for years. This shows that, in addition to environmental sampling, in some cases necropsies should be performed to confirm the *Salmonella* involved.

Using the results of farm sampling, further categorization of *Salmonella* status for each farm can be performed. If a very low number of samples are positive within a sampling period, the relevance for the farm must be discussed. This was the case for eight farms within this study, where only one environmental sample was positive for an exotic *Salmonella* serovar. In such cases, further sampling should be conducted prior to implementing a *Salmonella* control program. However, for farms with high positivity rates, especially in the nursery, control measures should be implemented. In our study, the median of positive samples within the nursery was 47.2% and the lower quartile was 16.7%, showing that most farms within the study need to implement control measures for *Salmonella*. Due to the high positivity rate in cleaned and disinfected compartments, the focus should be on the improvement of hygiene measurements. As STM was the predominantly detected serovar, in addition to hygiene measures, vaccination can help to reduce STM prevalence on-farm. Vaccination of sows using a live vaccine with a double attenuated STM strain (Salmoporc®, Ceva Santé Animale, Libourne, France) has been shown to reduce shedding around farrowing, and consequently reduce infection of piglets [9,24,47,63]. Vaccination of piglets leads to a reduction in STM shedding and prevalence at slaughter [64,65,66]. Autogenous vaccines, custom-made from the specific serovar(s) identified on the farm, can be employed when commercial vaccines are not available or effective against the *Salmonella* serovar of concern [67]. Except for vaccination, evidence of different serovars does not require different control measures. In addition to the implementation of strategies to reduce shedding of *Salmonella* by infected sows and piglets by feeding strategies [68], the use of acids [59,69] or improvement of housing [12], it is important to determine the residual sources of *Salmonella*, which could, for example, be contaminated feed/water pipes or rodents.

Within our study, no *Salmonella* could be detected on eight farms which underlines the results of a trial in the Netherlands, where it was possible to reduce the *Salmonella* prevalence to below the detection level [9]. As a follow-up, comparing the management systems of positive and negative farms could result in valuable information on the management risk factors with most impact on the *Salmonella* status of the farm.

In addition to the use in an initial evaluation of the status of the sow farms, sock and environmental swab samples are valuable in determining the success of implemented control measures [9,10,11]. Pooled fecal samples have already been shown to be more sensitive than individual samples, with 20 samples per pool identified as being the most sensitive indicator [37]. Socks taken within the pens were shown to be more sensitive than individual samples [11], aiding the demonstration of a successful reduction in the on-farm prevalence in longitudinal sampling parallel to implemented measures against *Salmonella* [9]. Previous studies have shown that the time needed to reduce *Salmonella* prevalence at farm level is months rather than weeks [9,24]; therefore, sampling intervals of every 3–6 months are sufficient. The monitoring of effective cleaning and disinfection should be performed on a regular basis and only requires the use of one sock and one swab sample per compartment.

## 5. Conclusions

In the present study, sock and environmental swab samples proved to be useful as non-invasive and easy-to-apply methods to screen sow farms for the presence of *Salmonella*, being suitable for all farm sizes. Our results underline the need to include breeding and multiplying levels into monitoring and control systems, as this allows the involved *Salmonella* serovars to be identified in early stages, before they are introduced into the fattening units and later into the food chain. Additionally, it could be underlined that there is a huge demand for thorough control of cleaning and disinfection to interrupt the *Salmonella* infection cycle. The use of sock and environmental swabs in combination proved to be crucial as the detection rates of each sampling method varied.

Sock and environmental swabs are useful for validating the implementation of *Salmonella* control measures. They can be used to verify the effects of various *Salmonella* reduction measures, such as cleaning and disinfection, optimizing walking routes, changing boots and vaccination [9,11].

## Figures and Tables

**Table 1:** Overview of samples (*n* = 2355) taken from different areas in 105 German sow farms (CI = confidence interval).

	Sock Swabs			Environmental Swabs			Total		
	Farms	Samples	Positive (%) 95% CI	Farms	Samples	Positive (%) 95% CI	Farms	Samples	Positive (%) 95% CI
Farrowing	104	358	73 (20.4) [16.3–25.9]	105	304	52 (17.1) [13.0–21.8]	105	662	125 (18.9) [16.0–22.1]
Nursery	104	522	289 (55.4) [51.0–59.7]	103	448	167 (37.3) [32.8–41.9]	105	970	456 (47.0) [43.8–50.2]
Gilt quarantine, insemination, gestation	75	235	62 (26.4) [20.9–32.5]	83	217	36 (16.6) [11.9–22.2]	84	452	98 (21.7) [18.0–25.8]
walkways	72	112	47 (42.0) [32.7–51.7]	52	80	19 (23.8) [14.9–34.6]	72	192	66 (34.4) [27.7–41.6]
Boot washers	0	0	0	15	15	10 (66.7) [38.4–88.2]	15	15	10 (66.7) [38.4–88.2]
Cleaned and disinfected compartments	23	29	9 (32.0) [15.3–50.8]	23	35	14 (40.0) [23.9–57.9]	23	64	23 (35.9) [24.3–48.9]
Total	104	1256	480 (38.2) [35.5–41.0]	105	1099	298 (27.1) [24.5–29.8]	105	2.355	779 (33.1) [31.2–35.0]

**Table 2:** Distribution of frequencies with McNemar test and Cohen’s Kappa values reflecting agreement between sock swabs and environmental swabs within the different sampled areas (farrowing, nursery, other areas).

Sampled Area		*p*-Value McNemar Test	Kappa
		Sock Swab	Environmental Swab
Farrowing	sock swabs		0.5951
	environmental swabs	0.8036	
Nursery	sock swabs		0.4000
	environmental swabs	<0.001	
other areas	sock swabs		0.3838
	environmental swabs	0.1516	

## Data Availability

Data are available from corresponding author upon request.

## Supplementary Materials

The following supporting information can be downloaded at: https://www.mdpi.com/article/10.3390/ani13061031/s1. Table S1: Individual results of Salmonella serovars detected on each farm within each area including information about farm size, type of farm, no. of samples and date of sampling.
