# Supplementary material for: Sock and Environmental Swabs as an Efficient, Non-Invasive Tool to Assess the Salmonella Status of Sow Farms
Source: Animals (Basel). 2023 Mar 11;13(6):1031. doi: 10.3390/ani13061031 (PMC10044664; doi:10.3390/ani13061031)
Supplement: Supplementary file 1 [file animals-13-01031-s001.zip › animals-2231648-supplementary.pdf]

farm	farm size	Herd type	date of sampling	no. samples taken	no. / (%) positive samles	farm status	serovars farrowing	other serovars farrowing	serovars nursery	other serovars nursery	serovars other areas	other serovars other areas
Salm1	1000	f-f	07.06.2016	8	1 / (12.5)	pos	n.d.	/	STM	/	n.d.	/
Salm2	2000	f-n	18.07.2016	23	5 / (21.7)	pos	SD	/	STM, others	SGC	others	SGC
Salm3	3000	f-f	27.06.2016	28	4/ (14.3)	pos	n.d.	/	STM, others	SGC	others	SGC, n.t.
Salm4	1200	f	13.11.2017	24	18 / (75.0)	pos	others	SGC, SGE	n.d.	/	SD, others	SGC, SGE
Salm5	300	f-f	08.12.2017	15	0 / (0.0)	neg	n.d.	/	n.d.	/	n.d.	/
Salm6	5000	f-n	24.12.2015	40	11 / (27.5)	pos	STM	/	STM	/	STM	/
Salm7	400	f-f	23.10.2015	16	9 / (57.1)	pos	others	SGC	others	SGC	STM	/
Salm8	6000	f-f	05.04.2016	31	13 / (41.9)	pos	STM, SD, others	SGC	STM, others	SGC	STM	/
Salm9	2800	f-f	18.05.2016	29	10 / (35.4)	pos	n.d.	/	STM, SD	/	STM, SD	/
Salm10	1200	f-n	19.10.2016	40	14 / (35.5)	pos	n.d.	/	STM	/	SD	/
Salm11	2000	f-n	14.03.2018	29	10 / (34.4)	pos	n.d.	/	STM	/	SD	/
Salm12	300	f-n	05.04.2017	32	6 / (18.8)	pos	n.d.	/	STM	/	n.d.	/
Salm13	300	f-f	17.05.2018	13	7 / (53.8)	pos	STM	/	STM	/	STM	/
Salm14	350	f-n	24.02.2017	21	3 / (14.3)	pos	n.d.	/	STM	/	n.d.	/
Salm15	1200	f-n	03.08.2017	24	8 / (33.3)	pos	others	SGE	n.d.	/	others	SGE
Salm16	130	f-n	24.07.2017	18	4 / (22.2)	pos	n.d.	/	STM	/	STM	/
Salm17	700	f-n	13.07.2017	40	29 / (72.5)	pos	others	SGE, n.t.	STM, others	SGE	SD, others	SGE
Salm18	300	f-f	05.03.2018	28	1 / (3.7)	pos	n.d.	/	STM	/	n.d.	/
Salm19	300	f-n	03.07.2018	26	14 / (53.8)	pos	STM, others	SGC	STM, others	SGC	STM	/
Salm20	250	f-n	01.08.2016	18	15 / (83.3)	pos	others	SGB, SGE	STM, SD	/	STM, others	SGB, SGE
Salm21	300	f-n	19.05.2016	14	5 / (35.7)	pos	others	n.t.	STM, SD	/	SD, others	n.t.
Salm22	150	f-f	05.09.2017	15	2 / (13.3)	pos	n.d.	/	n.d.	/	STM	/
Salm23	150	f-n	26.04.2019	16	5 / (31.3)	pos	n.d.	/	STM	/	n.d.	/
Salm24	400	f-n	03.11.2015	14	2 / (14.3)	pos	n.d.	/	STM	/	n.d.	/
Salm25	1200	f-n	09.05.2016	18	3 / (16.6)	pos	n.d.	/	STM	/	n.d.	/
Salm26	120	f-f	05.10.2017	24	11 / (45.8)	pos	STM	/	STM	/	STM	/
Salm27	1500	f-n	04.07.2016	28	10 / (35.7)	pos	SD, others	SGE	others	SGE	others	SGE
Salm28	800	f-n	15.07.2021	22	3 / (13.63)	pos	n.d.	/	others	SGC	STM, others	SGC
Salm29	300	f-n	19.10.2017	34	21 / (61.8)	pos	SD	/	STM	/	SD	/
Salm30	260	f-n	21.06.2017	34	7 / (20.6)	pos	STM	/	STM	/	STM	/
Salm31	600	f-n	22.11.2018	19	5 / (26.3)	pos	n.d.	/	STM	/	STM	/
Salm32	350	f-n	16.12.2015	9	3 / (33.3)	pos	n.d.	/	STM	/	STM	/
Salm33	540	f-f	02.06.2017	19	10 / (52.6)	pos	STM	/	STM, others	SGB	STM	/
Salm34	650	f-n	01.08.2016	18	14 / (77.7)	pos	STM	/	STM	/	STM	/
Salm35	300	f-f	12.12.2016	14	0 / (0)	neg	n.d.	/	n.d.	/	n.d.	/
Salm36	200	f-n	01.08.2016	18	11 / (61.1)	pos	n.d.	/	STM	/	STM	/
Salm37	200	f-n	30.05.2017	11	7 / (63.6)	pos	STM	/	STM	/	STM	/
Salm38	560	f-n	04.04.2018	19	6 / (31.6)	pos	SD	/	n.d.	/	SD	/
Salm39	130	f-f	19.12.2016	12	3 / (25.0)	pos	n.d.	/	n.d.	/	others	n.t.
Salm40	n.i.	f-f	23.05.2018	27	4 / (14.8)	pos	n.d.	/	STM	/	STM	/
Salm41	600	f-n	07.06.2016	25	17 / (68.0)	pos	n.d.	/	STM	/	STM	/
Salm42	120	f-n	04.04.2016	16	7 / (43.7)	pos	n.d.	/	STM	/	STM	/
Salm43	300	f-n	08.12.2016	30	3 / (10.0)	pos	n.d.	/	STM	/	n.d.	/
Salm44	180	f-f	22.02.2017	23	1 / (4.3)	pos	n.d.	/	STM	/	n.d.	/
Salm45	160	f-f	30.07.2019	22	2 / (9.1)	pos	n.d.	/	SE	/	SE	/
Salm46	200	f-f	15.02.2018	10	1 / (10.0)	pos	n.d.	/	n.d.	/	others	SE
Salm47	1600	f-n	27.02.2019	26	18 / (69.2)	pos	others	n.t.	STM, others	n.t.	others	n.t.
Salm48	450	f-n	29.11.2017	17	11 / (64.7)	pos	STM	/	STM	/	STM	/
Salm49	250	f-n	09.11.2018	23	6 / (26.1)	pos	n.d.	/	STM, SE	/	STM, SE	/
Salm50	230	f-n	22.11.2018	19	1 / (5.3)	pos	n.d.	/	n.d.	/	STM	/
Salm51	300	f-n	02.05.2017	32	2 / (6.3)	pos	n.d.	/	STM	/	n.d.	/
Salm52	1000	f-n	31.01.2017	50	19 / (38.0)	pos	others	SGC	STM, others	SGC	SD, others	SGC
Salm53	350	f-n	15.06.2020	24	10 / (41.6)	pos	STM, others	SGC	STM	/	n.d.	/
Salm54	130	f-f	23.05.2017	18	5 / (27.7)	pos	n.d.	/	n.d.	/	STM, others	SGE
Salm55	220	f-f	05.07.2017	15	4 / (26.6)	pos	n.d.	/	STM	/	STM	/
Salm56	150	f-n	22.05.2018	17	5 / (29.4)	pos	n.d.	/	STM	/	STM	/
Salm57	200	f-n	07.02.2018	19	0 / (0.0)	neg	n.d.	/	n.d.	/	n.d.	/
Salm58	400	f-n	19.01.2017	28	3 / (10.7)	pos	n.d.	/	STM	/	STM	/
Salm59	350	f-n	13.04.2016	22	13 / (59.1)	pos	n.d.	/	STM	/	STM	/
Salm60	120	f-f	27.03.2015	11	6 / (54.5)	pos	n.d.	/	STM	/	STM	/
Salm61	300	f-n	01.02.2016	22	4 / (18.2)	pos	SD	/	STM	/	STM	/
Salm62	800	f-n	01.12.2015	10	7 / (70.0)	pos	others	n.t.	others	n.t.	others	n.t.
Salm63	300	f-n	25.11.2015	31	4 / (12.9)	pos	n.d.	/	n.d.	/	STM, others	SGC
Salm64	580	f	30.03.2016	8	0 / (0.0)	neg	n.d.	/	n.d.	/	n.d.	/
Salm65	480	f-n	30.03.2016	10	2 / (20.0)	pos	others	SGE	n.d.	/	n.d.	/
Salm66	2000	f-f	20.01.2016	39	9 / (23.1)	pos	n.d.	/	STM	/	STM	/
Salm67	150	f-n	06.06.2016	16	1 / (6.3)	pos	n.d.	/	n.d.	/	SE	/
Salm68	300	f-f	28.06.2017	23	7 / (30.4)	pos	n.d.	/	SE	/	SE	/
Salm69	400	f-f	09.02.2017	20	2 / (10.5)	pos	SD	/	SD	/	n.d.	/
Salm70	1500	f-n	13.06.2018	28	15 / (53.6)	pos	STM, others	SGC	STM, others	SGC	others	n.t.
Salm71	n.i.	f-n	24.09.2018	22	7 / (31.8)	pos	others	n.t.	STM	/	SD	/
Salm72	100	f-f	22.05.2017	17	11 / (64.7)	pos	STM	/	STM	/	n.d.	/
Salm73	350	f-n	08.05.2017	20	5 / (25.0)	pos	n.d.	/	STM	/	STM	/
Salm74	400	f-n	06.11.2015	22	5 / (22.7)	pos	n.d.	/	STM	/	others	SGE
Salm75	80	f-f	25.02.2016	13	1 / (7.7)	pos	n.d.	/	STM	/	n.d.	/
Salm76	300	f-n	10.12.2015	15	0 / (0.0)	neg	n.d.	/	n.d.	/	n.d.	/
Salm77	100	f-f	23.02.2017	15	5 / (33.3)	pos	n.d.	/	STM	/	n.d.	/
Salm78	220	f-n	27.11.2015	11	6 / (54.5)	pos	SD	/	SD, SE	/	SD	/
Salm79	2500	f-n	07.07.2021	20	1 / (5.0)	pos	n.d.	/	n.d.	/	SD	/
Salm80	220	f-n	14.12.2017	20	7 / (35.0)	pos	n.d.	/	STM	/	STM	/
Salm81	150	f-n	21.10.2016	20	5 / (25.0)	pos	n.d.	/	STM	/	n.d.	/
Salm82	100	f-f	19.07.2017	25	7 / (25.9)	pos	n.d.	/	others	SGE	others	SGE
Salm83	n.i.	f-n	06.04.2016	26	12 / (46.2)	pos	STM	/	STM	/	n.d.	/
Salm84	280	f-n	19.02.2016	15	10 / (66.7)	pos	STM	/	STM	/	STM	/
Salm85	100	f-f	11.01.2016	21	0 / (0.0)	neg	n.d.	/	n.d.	/	n.d.	/
Salm86	200	f-n	28.02.2018	15	6 / (40.0)	pos	n.d.	/	STM	/	STM	/
Salm87	120	f-n	21.03.2017	32	13 / (40.6)	pos	n.d.	/	STM	/	STM	/
Salm88	400	f-n	18.06.2019	28	15 / (53.6)	pos	n.d.	/	STM	/	STM, others	n.t.
Salm89	350	f-n	13.04.2016	28	14 / (50.0)	pos	STM	/	STM	/	STM	/
Salm90	360	f-n	15.06.2018	26	3 / (11.5)	pos	n.d.	/	STM	/	n.d.	/
Salm91	1000	f-f	15.11.2017	22	2 / (9.1)	pos	n.d.	/	STM	/	n.d.	/
Salm92	3000	f-n	26.03.2019	19	8 / (42.1)	pos	others	SGC	STM, others	SGC	n.d.	/
Salm93	160	f-f	15.06.2018	10	2 / (20.0)	pos	n.d.	/	STM	/	n.d.	/
Salm94	230	f-n	04.07.2017	28	22 / (78.6)	pos	STM	/	STM	/	STM	/
Salm95	200	f-f	28.02.2016	14	8 / (57.1)	pos	SE	/	SE	/	SE	/
Salm96	250	f-n	21.12.2017	25	3 / (12.0)	pos	STM	/	n.d.	/	STM	/
Salm97	500	f-n	16.04.2018	21	0 / (0.0)	neg	n.d.	/	n.d.	/	n.d.	/
Salm98	650	f-n	20.09.2017	34	6 / (17.6)	pos	n.d.	/	STM, SD	/	SD	/
Salm99	1000	f-n	07.04.2017	46	22 / (47.8)	pos	SD, others	SGC, n.t.	SD, others	n.t.	SD, others	n.t.
Salm100	10000	f-n	28.02.2016	32	12 / (37.5)	pos	n.d.	/	STM	/	STM, SD	/
Salm101	50	f-f	27.02.2015	11	0 / (0.0)	neg	n.d.	/	n.d.	/	n.d.	/
Salm102	350	f-n	26.07.2017	19	2 / (10.5)	pos	n.d.	/	STM	/	n.d.	/
Salm103	500	f-n	18.02.2019	29	16 / (55.2)	pos	n.d.	/	STM, others	SGB	STM, SD	/
Salm104	10000	f-n	26.01.2015	46	21 / (42.9)	pos	STM, SD	/	STM	/	STM	/
Salm105	5000	f-n	16.12.2015	55	36 / (65.5)	pos	STM	/	STM	/	STM	/

Table S1:
Individual results of *Salmonella* serovars detected on each farm within each area including information about farm size, type of farm, no. of samples and date of sampling
(STM= *Salmonella* Typhimurium, SD= *Salmonella* Derby, SE= *Salmonella* Enteritidis, SGB= *Salmonella* Group B (without STM), SGC=*Salmonella* Group C, SGE=Salmonella Group E, n.t.=no typing, n.d.= no detection)
(n.i.= no information, f=farrowing farm, f-n= farrowing farm with nursery, f-f= farrow-finish)
